# Distribution, Population Dynamics, and Management of Moroccan Locust *Dociostaurus maroccanus* (Thunberg, 1815) (Orthoptera, Acrididae) in Tajikistan

**DOI:** 10.3390/insects15090684

**Published:** 2024-09-10

**Authors:** Khuramjon S. Khairov, Elena Lazutkaite, Alexandre V. Latchininsky

**Affiliations:** 1 E.N. Pavlovsky Institute of Zoology and Parasitology, National Academy of Sciences of Tajikistan, Dushanbe 734025, Tajikistan; khayrov.80@mail.ru; 2 TMG Research gGmbH, 10829 Berlin, Germany; 3 Locusts and Transboundary Plant Pests and Diseases, Plant Production and Protection Division (NSP), Food and Agriculture Organization of the United Nations (FAO), 00153 Rome, Italy

**Keywords:** Moroccan locust, *Dociostaurus maroccanus*, Tajikistan, pest management, population dynamics, agricultural impact, insecticides

## Abstract

**Simple Summary:**

The Moroccan locust (*Dociostaurus maroccanus*) has historically been a significant pest, causing substantial agricultural damage across Tajikistan. This study, conducted between 2012 and 2023, provides an overview of the locust’s distribution, population dynamics, and management in the country. We observe an increase in locust outbreaks within the period, with southern Tajikistan being the most severely affected. We emphasize the critical need for regional cooperation and sustainable management practices using environmentally friendly control agents.

**Abstract:**

This study contributes to the body of knowledge on the Moroccan locust (*Dociostaurus maroccanus*) in Tajikistan, exploring its distribution, population dynamics, economic significance, and management. Our research covers the period from 2012 to 2023. Over this period, there has been a documented increase in the size of the infested areas, with southern Tajikistan being the most severely affected. Outbreaks of economic importance happened each year throughout the timeframe. The total area impacted by infestations reached 752,130 hectares in southern Tajikistan alone. The paper places emphasis on the necessity of cross-border and regional collaboration and the adoption of environmentally sustainable control methods, such as the use of biopesticides.

## 1. Introduction

Locust outbreaks have plagued vast regions and affected millions of people worldwide since ancient times. For thousands of years, these outbreaks have posed significant challenges to farmers and livestock breeders, often leading to severe food shortages and famines in affected communities [1].

**Figure 1:**
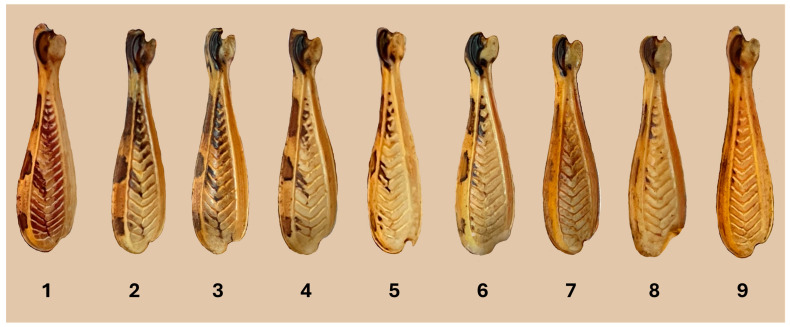
Development of dark spots on the external side of the right hind femur of adult Moroccan locusts; 1–3—solitary phase; 4–6—transiens (intermediate) phase; 7–9—gregarious phase. Collector: Khuramjon S. Khairov. Photo: Alexandre V. Latchininsky.

**Figure 2:**
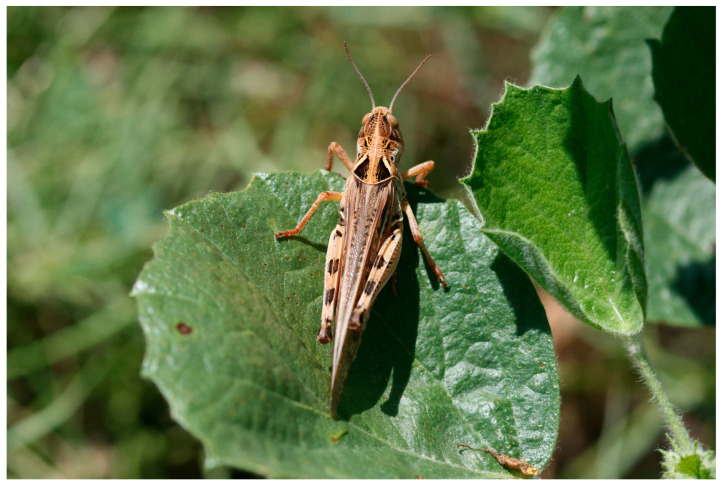
An adult *D. maroccanus* in the solitary phase. Photo: Alexandre V. Latchininsky.

**Figure 3:**
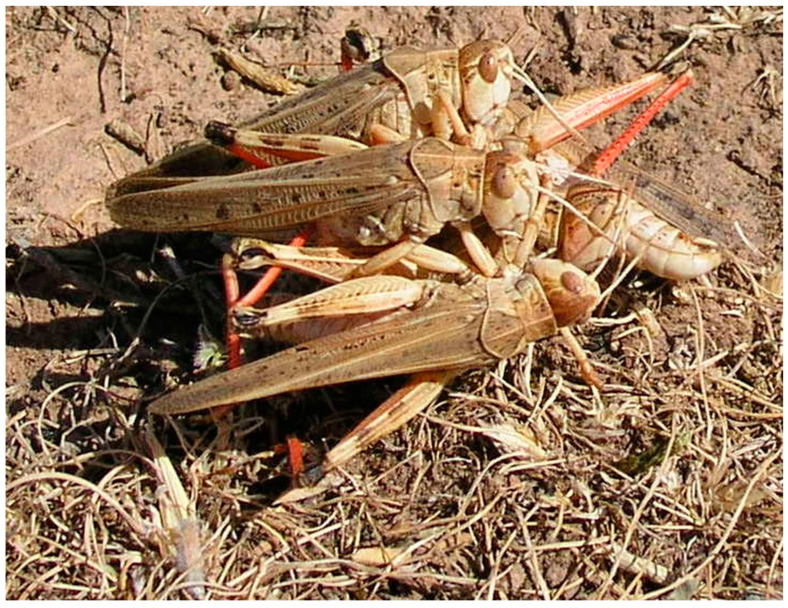
Adult *D. maroccanus* in gregarious phase with indistinct dark spots on outer side of hind femora. Photo: Alexandre V. Latchininsky.

The ability of locusts to pose such a significant agricultural threat is attributed to a phenomenon known as phase polyphenism. Globally, there are approximately a dozen species known to exhibit phase polyphenism [2]. These locust species, including the Moroccan locust *Dociostaurus maroccanus* (Thunberg, 1815)—the subject of this paper—possess a remarkable ability to undergo dramatic behavioral, morphological, and physiological changes shifting from a cryptic solitary lifestyle (solitary phase) to a gregarious phase [3] when their population density increases. B.P. Uvarov was the first to advance the hypothesis that *D. maroccanus* exhibits phase polyphenism in 1928 [4]. In 1932, S.P. Tarbinsky confirmed this hypothesis and described the main morphological and coloration differences between the solitary and gregarious phases of the Moroccan locusts [5]. For the adults, the distinctive features are summarized in Table 1. Changes in body coloration and size are thought to be adaptive for survival in dense, mobile groups. See Figure 1, Figure 2 and Figure 3 for color differences between adult *D. maroccanus* in solitary and gregarious phases.

The gregarious phase of locusts is marked by greater appetite, enhanced locomotor activity, and high mobility, initially in dense bands of marching nymphs and subsequently in expansive flying swarms of adults that can rapidly migrate across regions. These changes are mediated by neurohormonal pathways that are activated by the close physical presence of other locusts, in other words, when crowded [7].

Importantly, locusts switch to a gregarious phase as an evolutionary strategy to ensure their survival and expand their geographical range. Forming dense hopper bands and expansive swarms serves multiple purposes: it reduces individuals’ risk of being preyed upon by conspecifics and enables collective movement to areas rich in resources. The formation of large swarms also results in a significant increase in biomass, which overwhelms potential predators through “predator satiation”, thereby enhancing the survival chances of the swarm as a whole [8].

Recent studies on other locust species suggest that genetic and epigenetic mechanisms play a role in phase polyphenism. These mechanisms enable locusts to swiftly adapt their behavior and physiology in response to environmental cues like changes in population density. The role of epigenetic factors, including DNA methylation and histone modifications, in enabling rapid changes in gene expression without altering the underlying DNA sequence has been examined, as this mechanism allows for swift behavioral adaptations to environmental and climatic fluctuations [9]. In addition, the genetic basis of phenotypic plasticity in locusts, highlighting the influence of genetic variation on locust phase polyphenism and population dynamics, has been investigated. However, to date, there are no specific studies on the mechanisms of phase polyphenism in *D. maroccanus*.

### 1.1. Objectives of the Paper

Despite the Moroccan locust’s vast distribution area and notable impact as an agricultural pest, a significant knowledge gap, with a dearth of recent studies addressing its impact, phenological patterns, spread, and ecology, remains. This is particularly pertinent to *D. maroccanus* in Tajikistan. To illustrate, the only study that has provided an in-depth overview of *D. maroccanus* ecology in Tajikistan was published in Russian and dates back to 1937 [10]. Despite the fact that this country harbors important permanent breeding areas of the Moroccan locust, which provide connections to its other Central Asian habitats in Afghanistan, Kyrgyzstan, and Uzbekistan, research papers specifically focusing on *D. maroccanus* in Tajikistan are highly limited. Although, in 2023, a monograph by Latchininsky et al. [6] covered the species’ bioecology across most of its distribution range, including Tajikistan, it was published in Russian. The studies in English on *D. maroccanus* in Tajikistan are virtually nonexistent. This underscores the significant knowledge gap, especially in English-language publications.

Importantly, while the Moroccan locust undoubtedly threatens agricultural practices, its ecological significance in Tajikistan’s diverse landscapes, from plains to mountainous terrains, cannot be overlooked. The locust plays a pivotal role in the biogeochemical cycling of nutrients within the grassland ecosystem, facilitating the rapid recycling of substances sequestered in phytomass back to plant root systems [11]. The species also serves as a crucial trophic link in food webs, as a food source for various predators including birds, reptiles, amphibians, and other animals.

In this context, our paper presents an overview of the situation of the Moroccan locust in Tajikistan from 2012 to 2023. We identify outbreak locations and examine the yearly changes in infested areas across southern, central, and northern Tajikistan. Our study also provides insights into locust habitats, egg-laying sites, and phenology, particularly the timing of first hatching, which is important for the onset of management actions. Further, the paper covers population dynamics in southern Tajikistan (2014–2023). We also outline insecticide control interventions and explore collaborative governance strategies with neighboring counties. This is with a view to a balanced approach to locust management that considers the ecological importance of this locust and the need for effective and sustainable management practices to mitigate the impact on agriculture, food security, and the livelihoods of the affected rural populations.

### 1.2. Worldwide Distribution of the Moroccan Locust

The distribution range of *D. maroccanus* is fragmented, covering a vast longitudinal stretch of about 10,000 km. Starting from the west, it is found in the Atlantic islands (Madeira and Canary Islands) and extends eastward to Afghanistan and eastern Kazakhstan. In North Africa, it is prevalent in countries such as Morocco, Algeria, Tunisia, Libya, and Egypt. The species is also found on Mediterranean islands, including Corsica, Sardinia, Sicily, Cyprus, Crete, and Malta. In continental Europe, the Moroccan locust is found in the western and central regions, including Portugal, Spain, France, and Italy. It is also present in the Balkans, covering Bosnia and Herzegovina, Montenegro, Croatia, Macedonia, Slovenia, Serbia, Kosovo, Greece, and Bulgaria. Its northernmost habitats are in Hungary, Romania, Moldova, and Ukraine. Further to the east, in the Caucasus, *D. maroccanus* is found in Armenia, Azerbaijan, and Georgia as well as in southern Russia. In the Middle East and southwest Asia, the Moroccan locust’s presence is notable in Turkey, Syria, Lebanon, Israel, Jordan, Iraq, and Iran. Moving to Central Asia, the Moroccan locust is widespread, inhabiting Tajikistan—our country of focus—along with Turkmenistan, Uzbekistan, Kyrgyzstan, Kazakhstan, and Afghanistan [6]. Across its entire distribution range, the Moroccan locust is a univoltine species, completing a single generation per year [6].

### 1.3. Economic Importance of D. maroccanus

The Locust and Grasshopper Agricultural Manual [12] only assigns the highest “A” rank of economic importance, as a ‘major pest of many crops’, to a handful of locust species. *D. maroccanus* is one of those, together with the infamous desert locust *Schistocerca gregaria* (Forskål, 1775) and the migratory locust *Locusta migratoria* (Linnaeus, 1758). The gregarious phase (both hopper bands and swarms) can cause significant damage, devastating green vegetation, pastures, and crops. In the eastern regions of its geographic distribution, spanning the Caucasus and Central Asia (CCA), *D. maroccanus* is known to feed on over 210 plant species across 36 families, affecting more than 50 agricultural crops. This includes principal cereals, pulses, vegetables, forage, oil and industrial crops, as well as fruit trees and even conifers [13]. The Moroccan locust is recognized as the predominant locust pest in Azerbaijan, Uzbekistan, Kyrgyzstan, Turkmenistan, Iran, Afghanistan, and Tajikistan. Additionally, in the 21st century, *D. maroccanus* damage has been reported in 25 countries, including Spain, Italy (Sardinia), Turkey, Russia, Georgia, and Kazakhstan. Despite its high economic significance, there is a limited number of recent comprehensive studies on the Moroccan locust specific to CCA countries [14,15]. This paper aims to address this gap, focusing on Tajikistan.

### 1.4. Moroccan Locust in Tajikistan

**Figure 4:**
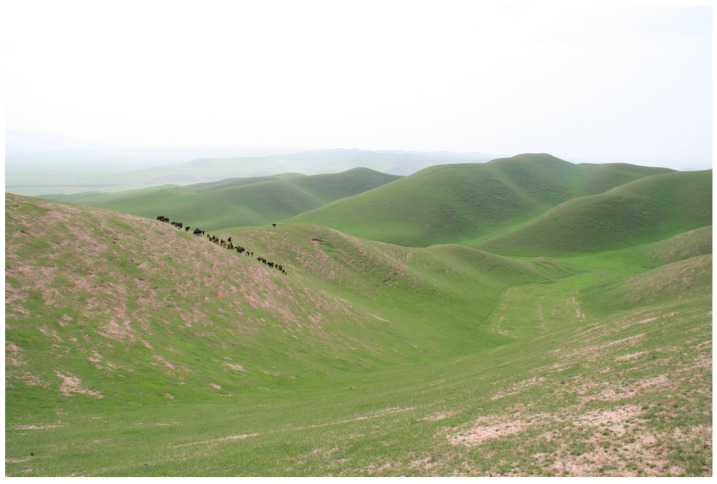
Topography of Moroccan locust habitats in Dangara district, Khatlon region southern Tajikistan. Photo: Alexandre V. Latchininsky.

**Figure 5:**
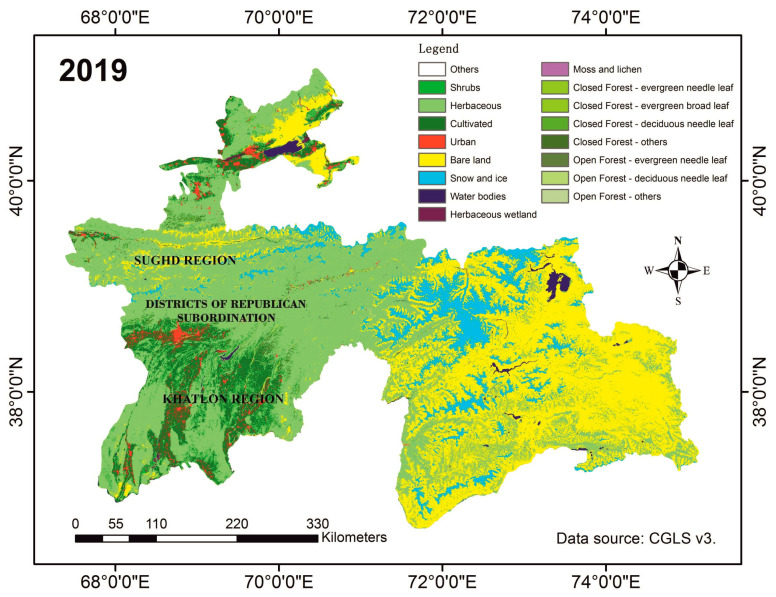
Landcover map of Tajikistan. Source: CGLS V3 2019 [20].

Tajikistan, located in Central Asia, has a diverse climate and is predominantly mountainous [16]. The Moroccan locust’s habitats are found in ecotones where two distinct ecosystems, foothills and plains (valleys), converge. Figure 4 illustrates the topography of the Moroccan locust’s habitats in the Dangara district of southern Tajikistan. Figure 5 illustrates the land cover. The topography in these habitats often includes open landscapes with limited shade, maximizing exposure to sunlight, which is beneficial for nymphal development. These transitional zones are characterized by warm temperatures (average annual air temperatures, ranging from 13.2 °C to 17.6 °C, and peak temperatures between 40.1 °C and 44.0 °C, low precipitation (typically below 250 mm per year), long dry periods, and ephemeral dry-steppe vegetation cover [6,10]. The vegetation cover in these transitional zones is dominated by short sedges (mainly *Carex pachystylis*) and grasses (primarily *Poa bulbosa*) [6,10]. Herbaceous vegetation in these zones provides crucial feeding grounds for locusts, supporting their life cycle and population dynamics (Figure 5). This distinctive combination of climate, terrain, and ephemeral spring vegetation creates an environment that is highly conducive to the reproduction of *D. maroccanus.*

Delving deeper into climatic and environmental factors, spring rainfall of about 100 mm from March to May plays a critical role in the population dynamics of *D. maroccanus* as it triggers rapid vegetation growth, providing essential food and habitat for locusts [6]. This lush growth, particularly following drought periods, often acts as a catalyst for population increase [6]. During droughts, the resulting sparse and patchy vegetation compels locusts to congregate in the few areas where food remains available. Such clustering, which contrasts with their typically dispersed solitary phase, can be accelerated by anthropogenic factors such as deforestation and overgrazing [17]. Intensive grazing can strip an area of green vegetation by early summer, exacerbating the clustering effect. This forces locusts to gregarize even more densely in the remaining patches of foliage.

In 20th-century Tajikistan, records concerning the geographic range and outbreak areas of *D. maroccanus* are sparse and poorly documented [10]. In the early 21st century, this locust emerged as a major pest in the southwestern regions of Tajikistan. Between 2006 and 2007, there was a marked surge in the locust population. In 2006, *D. maroccanus* outbreak affected both Tajikistan and Uzbekistan [18]. In 2007, the Khatlon region of southern Tajikistan and parts of central Tajikistan experienced an outbreak, in the form of dense hopper bands alongside adult swarms, affecting over 35,000 hectares and resulting in approximately Tajik Somoni (TJS) 18 million in damage; using conversion rates for 1 September 2007, this equates to USD 5.23 million [19].

Between 2008 and 2010, significant outbreaks of *D. maroccanus* were recorded in Uzbekistan’s Surkhandarya region, particularly along the Babatag Mountains bordering Tajikistan’s Khatlon region [14]. During the same period, the Moroccan locust situation in Tajikistan was serious, impacting multiple regions and prompting significant control efforts. In response, Tajikistan treated 73,400 hectares in 2010 and allocated approximately USD one million towards the activities under the newly created State Enterprise “Locust Control Expedition” (SE-LCE), Ministry of Agriculture. This allocation was directed towards surveying 350,000 hectares and conducting insecticide treatment campaigns on about 120,000 hectares in 2011. Since then, SE-LCE has been the main actor in locust monitoring and management in Tajikistan.

## 2. Materials and Methods

Aggregated national data on the infested and chemically treated areas between 2004 and 2023 were provided by the SE-LCE, Ministry of Agriculture. We conducted a linear regression analysis to determine if there was an increase in the size of infested areas over the period. The independent variable is the years, from 2004 to 2023, and the dependent variable is the areas infested. Additionally, the SE-LCE provided district-level data for southern, central, and northern Tajikistan covering the period from 2012 to 2023. Similarly, we conducted a linear regression analysis to determine if there was an increase in the size of infested areas within these districts over the specified period.

Following consultations with Tajik plant protection experts, this study defined an outbreak as an infestation affecting an area of 2000 hectares or more per district in Tajikistan. Although there is no officially recognized threshold, an area of 2000 hectares is considered to have significant economic implications in [21].

Observations of hatching were conducted under the limitations of resource constraints. The observation period covered the years from 2014 to 2024 in southern Tajikistan, while in central and northern Tajikistan, the observation period was confined to the years from 2021 to 2023. The prioritization of southern Tajikistan (Khatlon region) in this study was intentional, reflecting its highest susceptibility to locust infestations among the regions. Our study covers three administrative regions in Tajikistan: Khatlon in the south; Sughd in the north; and the Districts of Republican Subordination (DRS), located in central Tajikistan. Each of these is divided into multiple districts and each administrative district is divided into jamoats. We use the term ‘jamoat’ to refer to administrative and territorial subdivisions within Tajikistan. A jamoat represents a local government unit, akin to a rural municipality, encompassing one or more villages or communities. This definition aids in comprehending the administrative backdrop pertinent to our data gathering efforts.

In the course of the study, we collected over 2000 *D. maroccanus* specimens at various developmental stages. Each specimen was then classified into its respective stage, i.e., nymph of a particular instar, from 1 to 5, or adult, and densities and abundance were counted. For comparative purposes, the collection housed at the Institute of Zoology and Parasitology of the National Academy of Sciences was consulted. The materials were collected from the plain, steppe, and foothill–steppe zones of Tajikistan at elevations ranging from 450 to 1500 m above sea level. When selecting study sites, we considered vegetation type, substrate characteristics, topography, slope orientation (exposition), and elevation. We identified egg-laying sites and observed the hatching of nymphs from egg pods. The coordinates were determined using a Garmin eTrex H GPS device (Garmin Ltd., Olathe, KS, USA).

In southern Tajikistan, we counted the Moroccan locust abundance using a time-based insect counting method. This technique involved capturing insects with a standard entomological net (with a diameter of 40 cm) for a specified duration of time, usually 10 or 15 min. The results were then recalculated to represent a one-hour period, indicating the abundance (individuals per hour). We established the abundance counts for both nymphal and adult stages. The density of locusts (individuals per 1 square meter) was determined on transects, each comprising 25 sections of 1 square meter [11]. To count locust density, researchers walked each transect and recorded the number of individual locusts that jumped out of each of the envisioned, 1-square-meter areas. Our observations totaled 242, including 107 time-based recordings to assess abundance and 135 transect recordings to assess locust density. The abundance and density surveys took place during peak activity hours for locusts to maximize the detection probability. The observations were made under optimal weather conditions—sunny, warm, and devoid of strong winds. Notable limitations of this investigation were the limited observation size and the exclusion of meteorological data, which were not obtained due to the substantial time and financial resources required. Further studies should address these gaps. In addition, resource constraints and the COVID-19 pandemic years of 2020 and 2021 resulted in interruptions, affecting the completeness of our dataset.

Egg-laying sites were recorded with GPS and the number of egg pods per 1 square meter was calculated. The assessment of egg-pod density was conducted at the end of summer and at the end of winter. Soil samples (approximately one per hectare) were taken by digging out an upper layer of the soil (5–8 cm deep) from a 50 × 50 cm area. The soil was carefully sifted, and the egg pods were collected and placed in a container. The mean number of eggs per egg pod was identified. To calculate the density of egg pods per square meter, the number of egg pods found in a 50 × 50 cm sample was multiplied by four. In total, we collected 168 soil samples. We collected most of the samples, 156 in total, from southern Tajikistan, 11 samples from northern Tajikistan, and one sample was taken from central Tajikistan.

## 3. Results and Discussion

The study’s results are presented and discussed over four sections. First, we begin with *D. maroccanus* life cycle and ecology in Tajikistan. Following this, geographic distribution and outbreak areas are examined. The third section encompasses a study of population dynamics focused on southern Tajikistan. The final section focusses on the species’ impacts on agriculture, and control measures and governance aimed at mitigating these impacts.

### 3.1. Life Cycle and Ecology

#### 3.1.1. Hatching

In Tajikistan, the appearance of *D. maroccanus* hatchlings in early to mid-spring is determined by a combination of environmental factors and weather conditions. The hatching is nuanced due to the complex interplay between air and soil temperature, altitude, geographic location, and critically, the specific conditions of soil humidity and precipitation levels. The onset of the hatching process requires a combination of two factors: soil humidity, which results from about 100 mm of precipitation in the winter–early spring period; and the sum of degree days of about 130–155 (with a presumed threshold of 10 °C) [13]. As a result, the precise timing of hatching can fluctuate from year to year. Notably, the hatching of first-instar nymphs in the southern regions precedes that in the northern areas by several days or weeks, as detailed in Appendix A, Table A1 and Table A3.

In southern Tajikistan, in 2014 and 2015, the first appearance of hatchlings was recorded on the 20th and 24th of March, respectively. In the years 2016, 2018–2020, 2022, and 2023, hatching began between 3rd and 13th of March. In 2017, there was a delay in hatching, with the first hatchlings appearing on 6 April in the foothill regions of Khurasan. In 2021, the locust hatching in the Dangara and Panj districts of southern Tajikistan was noted as early as 20 February. Our most recent observations, as of the time of writing, in 2024, indicate that first hatchlings were observed on 23 February (see Table A1 in Appendix A). The observations from central Tajikistan between 2021 and 2023 indicate that hatching of *D. maroccanus* occurred between 14 and 18 March (see Table A2 in Appendix A). In northern Tajikistan, the observations between 2021 and 2023 recorded first hatching between 24 March and 20 April (refer to Table A3).

#### 3.1.2. Hopper Bands

**Figure 6:**
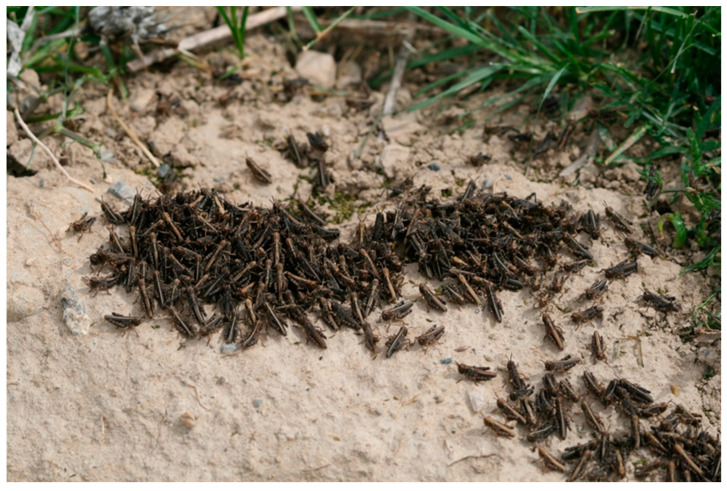
*D. maroccanus* early-instar nymphs in the gregarious phase. Photo: Alexandre V. Latchininsky.

In the solitary phase, locust nymphs are less likely to form large groups, but they may exhibit some level of aggregation in loose groups. This aggregation does not cause outbreaks and does not have an economic impact. In the gregarious phase, starting from the second instar, the nymphs exhibit gregarious behavior, forming dense bands known as hopper bands (see Figure 6). The formation of hopper bands is a critical early indicator of a developing outbreak and a precursor to the adult swarms.

The hopper bands are particularly noticeable in the mornings, when early-instar nymphs form dense “sun bands” to increase their body temperature in the rising sun [9]. The nymphal development period includes five instars and ranges between 25 and 40 days. This duration is influenced by environmental factors, primarily temperature. The maximum observed nymphal density in the first-instar hoppers reached an astonishing 21,000 individuals per square meter [22]. This is comparable with the data for the first-instar nymphal densities for the Australian Plague locust *Chorthoicetes terminifera* (Walker, 1870), 27,000 individuals per square meter, but less than in the Desert locust *Schistocerca gregaria* (Forskål, 1777), 37,000 individuals per square meter, and Asian Migratory locust *Locusta migratoria migratoria* (Linnaeus, 1758), 80,000 individuals per square meter [23]. Our highest recorded density was 1231 nymphs per square meter, observed in Dangara in 2019 (see Appendix A, Table A7).

To the naked eye, they often resemble the shadow cast by a cloud. During cooler morning temperatures, our measurements showed variability in the sizes of these locust bands, ranging from dimensions like 10 × 16 m to 7 × 24 m, and even as large as 33 × 11 m or 19 × 19 m. Additionally, we found the distance between these bands varied, ranging anywhere from 30 to 95 m apart. As temperatures rise throughout the day, the bands expand in size, and the nymphs increase their feeding activity. However, during peak heat, especially after 1 p.m. and at temperatures above 36 °C, the band tends to congregate closely, often on plants, with only a few nymphs continuing to feed [24].

As *D. maroccanus* progresses through developmental stages, the density decreases, while the occupied area expands and the smaller bands merge into bigger ones [25]. This territorial expansion becomes particularly pronounced in later developmental stages like fourth and fifth nymphal instars [26]. Fledging usually occurs in late spring or early summer, and mating begins 2 to 10 days later [2]. After fledging, winged adults form swarms; their density declines compared to younger nymphal instar stages, as the locusts begin to spread out over vast areas [4]. They move and expand to new regions for feeding and breeding. This movement pattern can have significant implications for agriculture and food security in affected areas.

#### 3.1.3. Oviposition

We observed that egg laying predominantly begins around mid-June. Notably, in the Dangara district of southern Tajikistan in the year 2022, the onset of oviposition was observed as early as 15 May. During the egg-laying phase, groups of Moroccan locusts can cover several hundred meters, presenting a distinct red–orange hue. Females typically deposit one or two egg pods during their lifespan, with occasional observations of up to four egg pods. Each egg pod contains an average of 30–35 eggs, with the range varying from 16 to 40 eggs [4]. Our observations in southern Tajikistan revealed considerable variations in the density of egg pods, with some containing as many as 39 eggs. The highest density we recorded was in Jayhun (southern Tajikistan) in 2017, with up to 3416 egg pods per square meter (see Appendix A, Table A7). From the literature, the highest Moroccan locust egg-pod densities are known to reach 8000 per square meter in Uzbekistan [11]. Of note, the variations in the number of eggs per pod and rate of pod production between the gregarious and solitary phases have not been comprehensively studied in *D. maroccanus.* Considering that females in the gregarious phase are larger than those in the solitary phase, it is plausible that they could produce more eggs.

**Figure 7:**
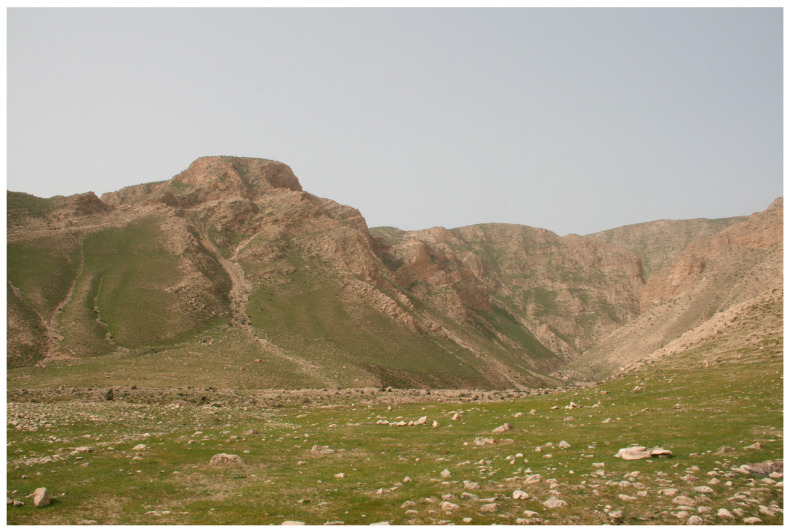
Flat rocky terrain which serves as oviposition site for *D. maroccanus* in Nosiri Khusrav district, Khatlon region, southern Tajikistan. Photo: Alexandre V. Latchininsky.

Notably, *D. maroccanus* exhibits specific preferences for its oviposition sites and seeks out undisturbed areas. Our observations indicate that their preferred sites are predominantly located in open steppes, hilly landscapes, rocky terrains, and foothill zones, with a particular inclination towards eastern foothill slopes and flat terrains (Figure 7). They were consistent across multiple years and various locations within Tajikistan.

The soil’s characteristics are an important limiting factor. In this respect, the Moroccan locust is different from the Italian locust *Calliptamus italicus* (Linnaeus, 1758), another locust species of economic importance in northen Tajikistan (Sughd region). The Italian locust does not have strong preferences for a particular soil for oviposition and can lay eggs in virtually any substrate, from light sandy soils to cropland areas or even cracks in the road asphalt. In Tajikistan, *D. maroccanus* favors dry, dense, compact, slightly saline soil with a high clay content, typically comprising 58% sandy, 9% clayey, and 33% loamy particles [6]. Occasionally, oviposition sites may also feature fine rocky soil (see Figure 7). Our observations showed that the oviposition sites are typically interspersed with patches of bare soil and xerophytic ephemeral vegetation, predominantly from the sedge (*C. pachystylis*) and grass (*P. bulbosa*) families. This mosaic of bare soil and vegetation patches creates an ideal environment for both oviposition and subsequent nymphal feeding.

In comparison to the mid-20th century, the 21st century has seen a notable upward shift of approximately 200–300 m in the upper limit of the Moroccan locust’s distribution, a change likely linked to climate warming. Rising temperatures have caused ephemeral plant formations and vegetation belts to gradually shift to higher altitudes [27]. In the 20th century, elevations between 460 and 600 m above sea level have been most common for *D. maroccanus* egg laying [28]. However, recent observations indicate oviposition at altitudes up to 1500 m and even higher in Central Asia [13]. In our research, the highest recorded elevation for oviposition was 1080 m above sea level, observed in the Dangara district of the Khatlon region in south Tajikistan (Table A7).

The life cycle of *D. maroccanus* concludes with the adults dying off in mid-summer, whereas the embryonic period is the longest in the life cycle as it lasts between approximately nine and ten months, from mid-summer to early spring [5,23,24]. Embryonic development starts immediately after the oviposition but after several days it stops, presumably under the influence of extremely high summer temperatures [29]. It resumes late in the autumn for several weeks but then stops again with eggs entering the winter diapause [30]. The embryonic development resumes in late winter–early spring under appropriate temperature and humidity conditions, leading to hatching.

### 3.2. Geographic Distribution and Outbreak Areas

**Figure 8:**
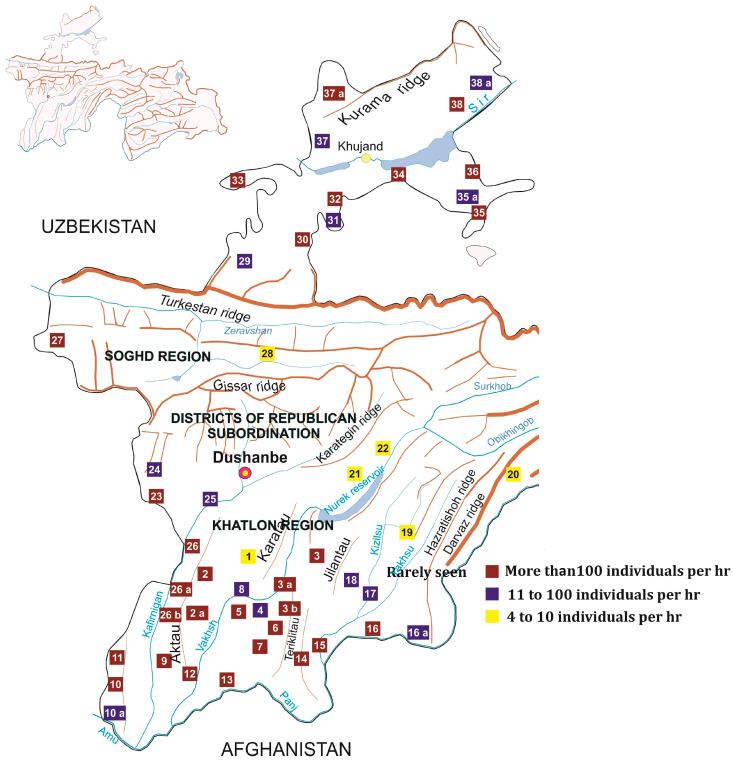
Geographic distribution of *D. maroccanus* populations across Tajikistan by density. Locations: 1—Yavanska Valley; 2a—Khurasan District; 3a,b—Dangara District; 4—Levakant; 5—Kushaniyan; 6—Vakhsh; 7—Badhi Jamoat; 8—A. Jomi; 9—Kabadiyan; 10a—Shahritus; 11—N. Khusrav; 12—Dusti; 13—Jayhun; 14—Pyanj; 15—Farkhor; 16a—Hamadoni; 17—Vose; 18—Temurmalik; 19—Muminobod; 20—Darvoz; 21—Fayzobod; 22—Ragun; 23—Tursunzoda; 24—Shahrinav; 25—Gissar; 26a,b—Rudaki; 27—Panjakent; 28—Aini; 29—Istaravshan; 30—Devashtich; 31—Spitamen; 32—Rasulov District; 33—Zafarobod; 34—B. Gafurov District; 35a—Isfara; 36—Konibodom; 37a—Mastchokh; 38a—Asht.

Figure 8 depicts the geographic distribution of *D. maroccanus* in Tajikistan, highlighting different densities of the species. Outbreak locations are marked in dark red (locusts found in mass with more than 100 individuals found in one hour of collection). Areas where the locust is frequently found (11 to 100 individuals per hour of collection) are marked in dark blue. Areas with scattered solitary populations (4 to 10 individuals per hour of collection) are marked in yellow.

Furthermore, there is evidence of locust migration from and to neighboring countries, specifically Uzbekistan, Kyrgyzstan, and Afghanistan. The relevance of these migratory patterns is underscored by the lengths of Tajikistan’s borders with these countries: the Tajik–Afghan border stretches 1357 km, the Tajik–Kyrgyz border 972 km, and the Tajik–Uzbek border 1312 km. In other parts of the Republic, the Moroccan locust is mostly observed in its solitary phase and at economically insignificant densities.

#### 3.2.1. Southern Tajikistan

In southern Tajikistan, the Moroccan locust’s primary habitats and outbreak areas are identified in the districts of Khurasan, Dangara, Vakhsh, Dusti, Jayhun, Pyanj, and Farkhor of the Khatlon region (refer to Appendix A, Table A4 for data on the infested areas across districts of southern Tajikistan). Overall, across the 12-year period, every year saw the gregarious phase of *D. maroccanus*. The data demonstrate that every year multiple districts experienced outbreaks of *D. maroccanus*, as defined by having infested areas of 2000 hectares or more. The total area affected by infestations reached 752,130 hectares over the 12-year period, marking the Khatlon region as the most heavily impacted by the infestations (Table A4). The years 2022 and 2023 saw the largest affected areas at 79,482 and 81,951 hectares, respectively.

**Figure 9:**
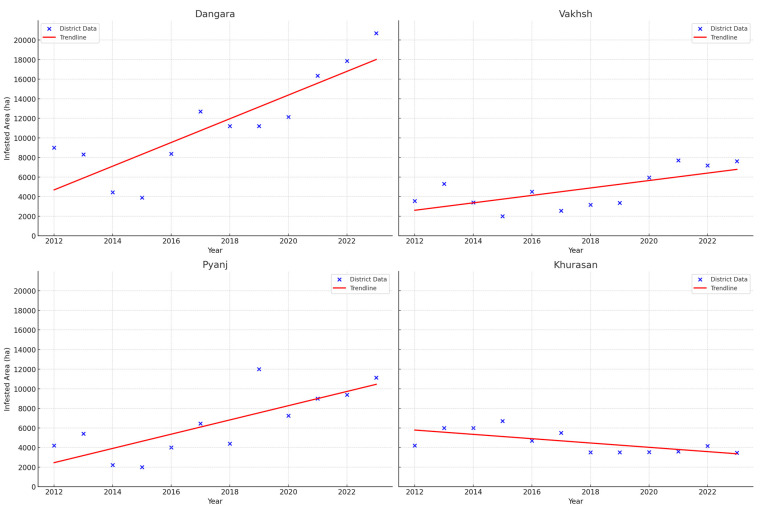
Areas infested by Moroccan locust in Dangara, Vakhsh, Pyanj, and Khurasan districts (Khatlon region), 2012–2023.

We observed the locusts predominantly inhabiting the foothill pastures of the Aktau, Tereklitau, Karatau and Sanglok ridges, typically at elevations ranging from 480 to 1200 m above sea level. The Sanglok ridge’s eastern spurs and the Tereklitau ridge’s eastern and southern spurs, particularly within the Dangara and Pyanj districts have the most widespread Moroccan locust distribution. Each district’s infestation data were analyzed using linear regression. Dangara, Pyanj, and Vakhsh districts showed a significant increase in infested areas over the time frame, as indicated by *p* < 0.01 (Dangara: *p* = 0.001, R^2^ = 0.737; Pyanj: *p* = 0.002, R^2^ = 0.616; Vakhsh: *p* = 0.015, R^2^ = 0.462;). Khurasan was the only district showing a negative trend in infested areas (*p* = 0.017; R^2^ = 0.452). These findings help identify which districts should be prioritized for monitoring, targeted interventions, and further research (see Figure 9).

#### 3.2.2. Central Tajikistan

**Figure 10:**
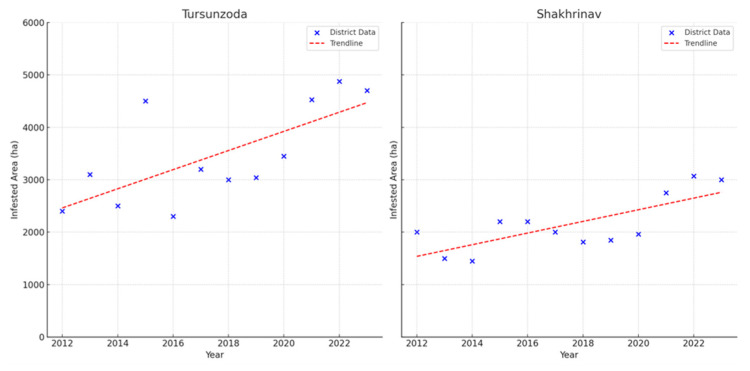
Areas infested by Moroccan locust in Tursunzoda, and Shakhrinav districts (Districts of Republican Subordination), 2012–2023.

In comparison to southern and northern Tajikistan, central Tajikistan provides fewer suitable habitats for locusts and experiences smaller outbreaks. In the center of Tajikistan, Moroccan locust outbreaks were noted in the Districts of Republican Subordination such as Tursunzoda, Shakhrinav, Gissar, and Rudaki. As detailed in Appendix A, Table A5, there are discernible fluctuations in infestation levels over years. Rudaki has experienced the highest cumulative infestation over the period, followed by Tursunzoda. The years 2021, 2022, and 2023 recorded the largest affected areas, similar to the observations in Tajikistan’s southern regions. Throughout the 12-year timeframe, every year showed an outbreak in at least two districts, using a threshold of infestations larger than 2000 hectares. The total area affected by infestations in Central Tajikistan amounted to 139,764 hectares (Table A5). Figure 10 presents that Tursunzoda, and Shakhrinav show a significant increase in infested areas over time (Tursunzoda: *p =* 0.011, R^2^ = 0.490; Shakhrinav: *p =* 0.005; R^2^ = 0.563).

#### 3.2.3. Northern Tajikistan

**Figure 11:**
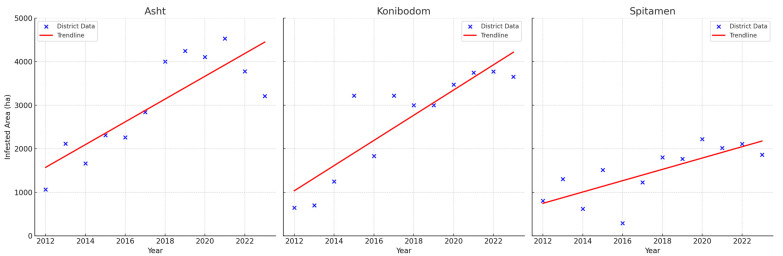
Area infested area by Moroccan locust in Asht, Konibodom, and Spitamen districts (Sughd region), 2012–2023.

In the Sughd region, the years 2013, 2017, and the period from 2019 to 2023 mark the largest outbreaks across multiple districts. Temporally, the peak in infestations from 2019 through 2023 aligns with the observations in central and southern Tajikistan. Over the twelve-year period, every year witnessed outbreaks in at least four districts. The combined infested area reached 434,667 hectares, which is three times more than in the central region (see Appendix A, Table A6). Figure 11 shows the trend in Asht, Konibodom, and Spitamen districts shows a significant increase over the twelve- year period (Asht: *p* = 0.001, R^2^ = 0.691; Konibodom: *p* = 0.001, R^2^ = 0.773; Spitamen: *p* = 0.005, R^2^ = 0.567). 

### 3.3. Moroccan Locust Population Dynamics in Southern Tajikistan (2014–2023)

This section presents field observations of Moroccan locust population dynamics in southern Tajikistan from 2014 to 2023. We observed the Moroccan locust egg-pod density, nymph, and adult density and abundance across twelve districts in southern Tajikistan (detailed in Table A7). For *D. maroccanus*, nymphal densities exceeding 5 per 1 square meter indicate the gregarious phase and are considered above the economic threshold in Tajikistan. For example, a nymphal density of 1–4 per square meter in 2019 in N. Khusrav district indicates the solitary phase. It is important to note that although specific observation sites have solitary populations, and thus, low densities, the district may experience an outbreak outside our observation areas. To accurately identify an economically significant outbreak, one needs to assess infested areas at both the district and regional levels. The following section presents the results, focusing on southern Tajikistan by year, starting with 2014.

In early April 2014, within the Vakhsh Valley, specifically in the Vakhsh district’s Mashaal jamoat, the density of egg pods was recorded to be between 21 and 37 per square meter and nymphal density ranged between 30 and 117 per square meter. The average nymph count was estimated at about 7400 nymphs per hour. The infested area in Vakhsh remained relatively low, spanning 3400 hectares.

At the beginning of April 2015, a rise in the species’ population was observed in the Vakhsh Valley, with counts reaching 17,192 nymphs per hour. The egg-pod density ranged from 31 to 40 per square meter, and nymphal density varied between 80 and 350 per square meter. In a different section of the valley, near the Kushaniyan river in the Bustonkala jamoat (Kushaniyan district), an outbreak was identified in mid-April 2015. This area is characterized by its flat steppe patches interspersed with hills; the recorded egg-pod densities were 18 to 25 per square meter. Nymphal densities ranged between 93 and 423 per square meter, with the nymph count peaking at 13,435 individuals per hour. Within the northern part of the Aktau ridge, specifically in the Khurasan district’s Kizilkala jamoat, we identified locust oviposition sites and observed an outbreak. In this region, the count of egg pods varied between 28 and 46 per square meter. Nymphal density ranged from 65 to 183 individuals per square meter, with an average abundance reaching up to 11,969 individuals per hour. In early June 2015, widespread mating and oviposition were documented in the Kizilkala jamoat. The density of locusts fluctuated considerably, ranging from 2 to 25 adults per square meter, with the average count reaching 3246 adults per hour. The infested area in Khurasan amounted to 6700 hectares. In the southwestern foothills of the Tereklitau ridge, specifically within the Jayhun district’s Ozodi-2 jamoat, we observed varying locust densities in the plain–steppe patches. Densities ranged between 5 and 47 adult locusts per square meter, whereas the average adult abundance count reached up to 6420 specimens per hour. In the arid, short-grass pastures, particularly on the flat stretches between hills, locusts were spotted during their mating activities. Jayhun recorded the largest affected area in southern Tajikistan, amounting to 7800 hectares.

In 2016, high population surges were identified in the J. Balkhi, Dusti, Khurasan, Jayhun, Dangara, Farkhor, and Vakhsh districts. In Dangara, nymphal abundance reached 14,346 individuals per hour, in Dusti 11,138 individuals per hour, and in Jayhun 11,042 individuals. Between late March and mid-April, we observed a substantial presence of gregarious third- and fourth-instar nymphs in the districts of Khurasan, Jayhun, Vakhsh, and Kushaniyan.

Within the study period, 2017 stood out as a challenging year for the Moroccan locust infestations in southern Tajikistan. Dangara district alone recorded 12,700 hectares affected, contributing to the region’s total of 75,831 hectares. Khurasan and Jayhun districts recorded astonishing counts of 2400 and 3416 egg pods per square meter, respectively.

In 2018, in the Khurasan district, nymphal densities varied from 15 to 413 per square meter, whereas the nymph count per hour reached 12,532 specimens. In Dangara, nymphal densities fluctuated between 32 and 398 per square meter, with the average abundance reaching 10,986 individuals per hour. The infested area in Dangara remained extensive, covering around 11,200 hectares.

In 2019, in Dangara, nymphal densities escalated to 1231 per square meter, with an average count of 12,300 nymphs per hour and 11,195 hectares infested. As previously noted, field observation efforts in 2020 and 2021 were impeded by COVID-19 restrictions. Nevertheless, the reported infested areas in the region continued to be substantial and constituted economically significant outbreaks. Specifically, Dangara recorded infestations covering 12,134 hectares and 16,350 hectares in 2020 and 2021, respectively.

The Khatlon region experienced its highest levels of infestation in 2022 and 2023. In 2022, areas of high locust density and egg laying were identified in Farkhor, Vakhsh, Dangara, and Pyanj districts. Farkhor recorded nymphal densities of 43 to 332 per square meter and a high average abundance count of 14,627 nymphs per hour. In Dangara, the observed average adult count reached 8214 specimens per hour, with the infested area expanding to 17,865 hectares.

**Figure 12:**
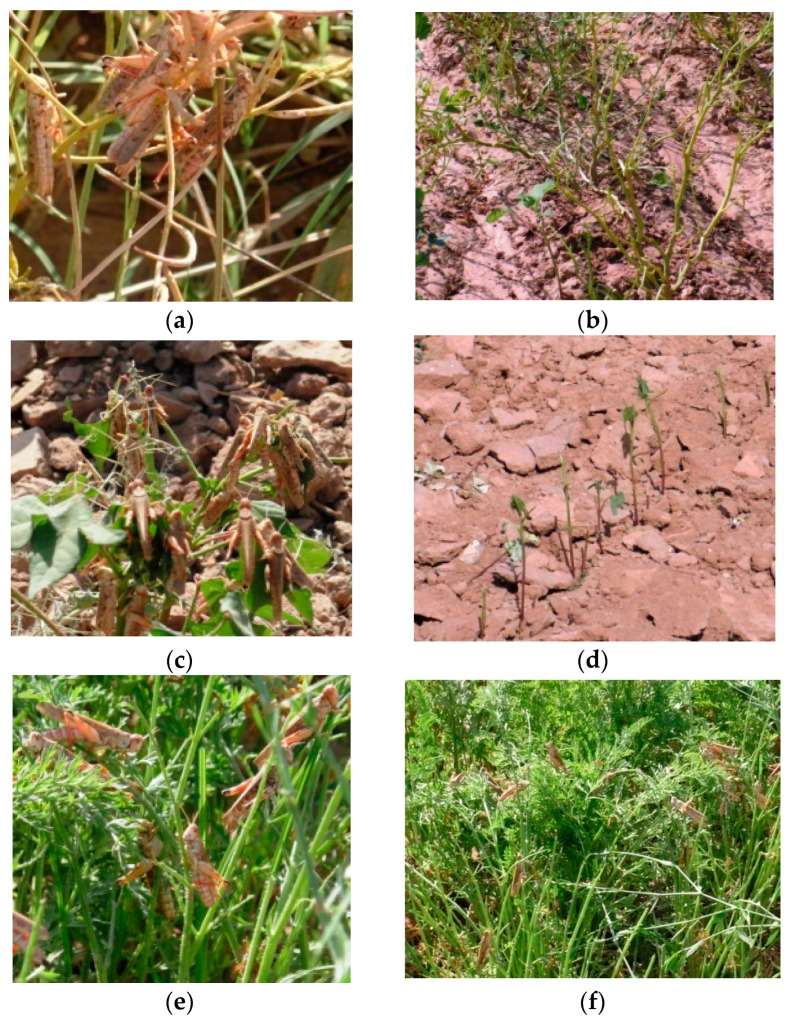
Moroccan locust damage to agricultural crops: (**a**,**b**) legumes; (**c**,**d**) cotton; (**e**,**f**) carrots. Photos: Khuramjon Khayrov.

In early June 2023, a notable increase in Moroccan locust infestations was observed in the rural areas of the Dangara district, especially within the Lolazor jamoat, as well as in the Hamadoni district (Dashtigulo jamoat) and the Farkhor district (Zafar, Somonjon, Navabadi Bolo, and Navabadi Poyon jamoats). The average adult count in Dangara reached 6934 specimens per hour, and in Hamadoni, it was 7323 adults per hour, highlighting ongoing control challenges. The infested area in Dangara peaked at record 20,700 hectares. These regions experienced considerable damage to various crops including vegetables, melons, industrial crops, grains, and forage, as depicted in Figure 12. This year marked one of the most severe Moroccan locust outbreaks in Tajikistan in the 21st century, with a total of 81,951 hectares infested in the southern part of the country.

### 3.4. Moroccan Locust Control: Its Implications and Governance

As in other locust-affected countries, in Tajikistan, monitoring is the basis of management actions. Locust monitoring includes four separate surveys conducted at different times of the year carried out by SE-LCE.

**Figure 13:**
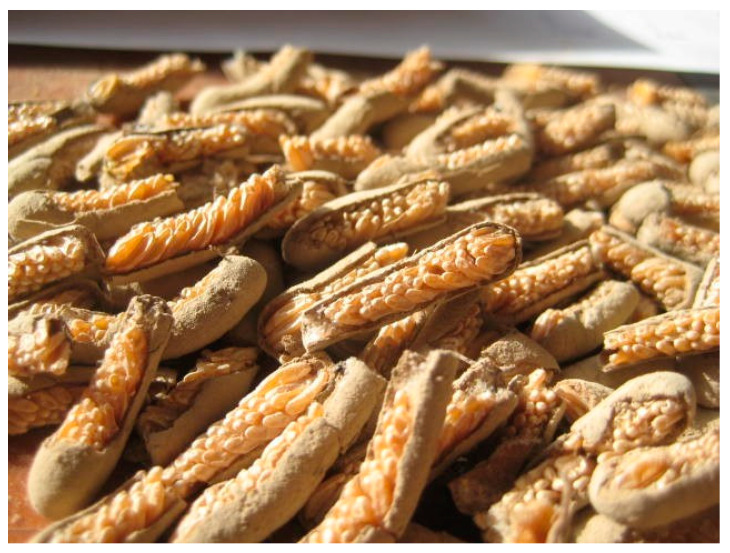
Egg pods of the Moroccan locust with partially removed walls, allowing examination and egg counting. Photo: SE-LCE.

1. Survey of adults during mass egg laying. This is the most important survey, with an objective to find and register egg-laying sites (egg beds). Based on the results of this survey, a preliminary forecast of the next year’s infestations is developed. It is conducted approximately one or two weeks after mass fledging. The survey is performed by scouts taking samples along the predetermined transects. The distance between parallel transects is 100–200 m, the distance between samples is 100–200 m. The sample is taken by approaching a visually delimited one-square-meter plot and counting the number of insects which jump out of it. Also, adults are collected to identify phase and egg development.
2. Summer/autumn egg-pod survey. Its objective is to identify areas infested by egg pods. Based on the results of this survey, the forecast of the next year’s infestations is finalized. It is conducted in the summer/autumn, after the end of the locusts’ annual cycle. Itineraries/transects are performed similarly to the previous survey. Soil samples (approximately one per hectare) are taken by digging out an upper layer of the soil (5–8 cm deep) from a 50 × 50 cm area. The soil is carefully sifted, and the egg pods are collected and placed in a container. The mean number of eggs per egg pods is identified, as well as the percentage of egg pods damaged or destroyed by predators and diseases (Figure 13). To calculate the density of egg pods per square meter, the number of egg pods found in a 50 × 50 cm sample is multiplied by four.
3. Spring partial egg-pod survey. Its objective is to assess the overwintering of eggs. Based on the results of this survey, the forecast of infestations is adjusted. It is conducted before hatching, usually in January/February or early March. The methodology is similar to the summer/autumn egg-pod survey but it is performed selectively, on 10% of the infested area, approximately one sample per 10 ha. The percentage of egg pods damaged by predators or infections is calculated.
4. Nymphal survey during hatching. Its objective is to verify the areas infested by nymphs, which are subsequently subject to control. This survey is the basis for anti-locust treatments. It is conducted during mass hatching of hoppers, in February–March. The methodology is similar to the adult survey, transects are 100–200 m apart and samples are spaced 100–200 m apart too. Nymphal density (number of hoppers jumping out of 1-square-meter plot) and developmental stage (by collecting hoppers with a net and identifying instars by examining their wing pads) are assessed.

The results of each survey are entered into an automated system of data collection (ASDC) developed by the Food and Agriculture Organization of the United Nations (FAO) in the framework of its “Programme to improve national and regional locust management in Caucasus and Central Asia”, operational since 2011 in ten CCA countries. ASDC is an app working on smartphones of tablet computers, its data are fed into the Caucasus and Central Asia Locust Management System (CCALM), a Geographic Information System also developed by FAO. CCALM facilitates the analysis of locust data as well as forecasting and early warning.

In Tajikistan, the chemical control method has been the ‘conventional’ approach against the Moroccan locust and other economically significant acridid species [24,25]. Once the spring nymphal surveys identify areas infested with densities exceeding the economic threshold of 5 nymphs per square meter, these areas become subject to treatment with insecticides. Typically, all such areas are subsequently treated, therefore treated areas can serve as an approximation of the infested areas. The primary insecticides used are pyrethroids and, less frequently, organophosphates, specifically, the water-based formulations containing 10% alpha-cypermethrin, 5% lambda-cyhalothrin, and a combination product containing 50% chlorpyrifos and 5% cypermethrin. The choice of insecticide depends on the locust’s developmental stage [2]. Insecticides such as 10% alpha-cypermethrin and 5% lambda-cyhalothrin target nymphal stages, while a combination product containing 50% chlorpyrifos and 5% cypermethrin is typically used during the adult phase post-fledging. The latter situation can result from immigration transborder swarm flights from adjacent countries, which requires crop protection treatments with fast-acting insecticides.

**Figure 14:**
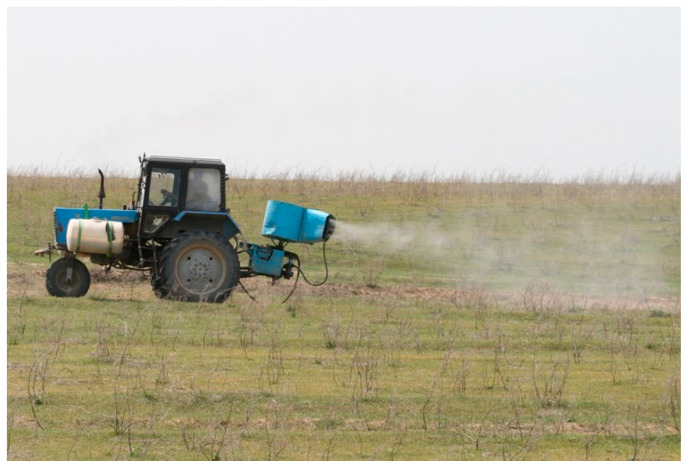
Spraying Moroccan locust infestations with tractor-driven sprayer OVH-600. Photo: Alexandre V. Latchininsky.

**Figure 15:**
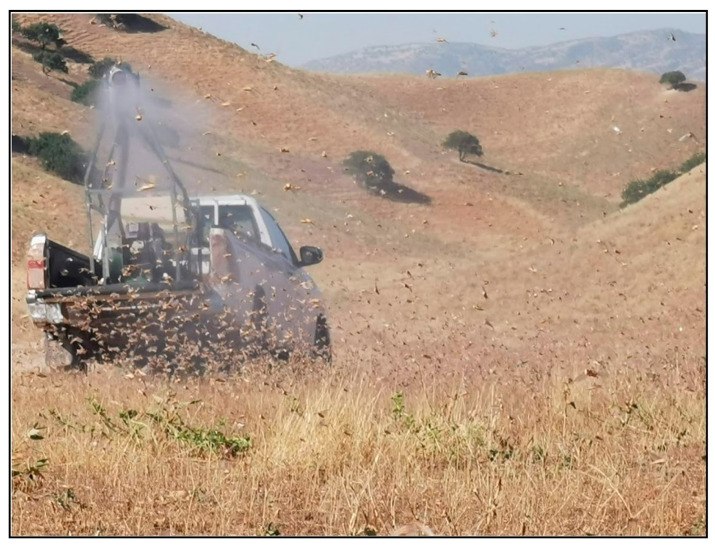
Treating a swarm of adult Moroccan locusts with the vehicle-mounted ULV sprayer Micron AU-8115. Photo: Oleg T. Guchgeldyev.

The equipment used for spraying broad-spectrum chemical insecticides ranges from handheld devices and knapsack atomizers, such as MicroUlva+ (Micron Group, Bromyard, Herefordshire, UK) and AU-8000 (Micron Group, Bromyard, Herefordshire, UK), to tractor- and vehicle-mounted sprayers like OVH-600 (AO Agregatnyj Zavod, Tashkent, Uzbekistan) (Figure 14), Agromaster TOS-2000 (Agromaster, Konya, Turkey), Tifone (Tifone Ambiente Srl, Modena, Italy), and Micron AU-8115 (Micron Group, Bromyard, Herefordshire, UK) (Figure 15). Although the country possesses a large number of ultralow-volume (ULV) sprayers designed to apply oil-based formulations, and thus, capable of treating locust infestations without using any water, these sprayers are mostly used to apply water-based insecticides. Water is a scarce commodity in many remote and mountainous locust-infested areas in Tajikistan. Therefore, it is crucial for the country to transition from using water-based insecticides in full volume, which require between 200 and 400 L per hectare, to spraying oil-based insecticide formulations in ULV mode, at a rate of just one liter per hectare. This shift will allow the locust control unit, SE-LCE, to fully benefit from the ULV technology developed specifically for arid regions suffering from water deficits.

**Figure 16:**
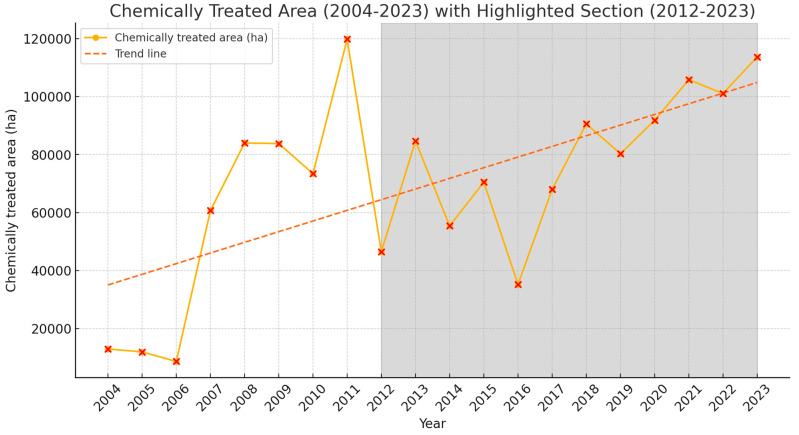
Chemically treated area (2004–2023): Annual treated area (ha), with a linear regression trend line indicating an upward trajectory. The shaded region demarcates the period 2012–2023.

The areas treated annually against the Moroccan locust in Tajikistan are illustrated in Figure 16 for the period 2004–2023 (see also Table A8). The trend line, derived from the linear regression analysis, demonstrates a positive slope, suggesting an annual rise in treated hectares between 2004 and 2023 (*p* = 0.002; R^2^ = 0.435). Focusing on the specific timeframe of 2012–2023, the data also reveal a significant upward trend (*p* = 0.003; R^2^ = 0.612).

Between 2012 and 2023, the cumulative area treated against the Moroccan locust in Tajikistan reached a staggering 943,636 hectares, which equals an annual average of 78,636 hectares treated chemically. In 2023, in the Khatlon region (southern Tajikistan) alone, insecticide treatments spanned 78,801 hectares. In the Districts of Republican Subordination (central Tajikistan), 17,475 hectares were treated, and in the Sughd region (northern Tajikistan), the area sprayed was 17,405 hectares. Altogether, the chemical insecticide treatment campaigns against the Moroccan locust encompassed a total of 113,681 hectares nationwide in 2023.

It is worth noting that the area treated annually against *D. maroccanus* in Tajikistan during the study period is higher compared to the 20th century, when they seldom exceeded 60,000 hectares per year [6]. A notable exception occurred in 1933, when, following a massive swarm invasion from Afghanistan and subsequent egg laying on an area of 138,000 hectares in 1932 [6], the extent of chemical control surged to an unprecedented 330,692 hectares. This example illustrates the interconnectedness of the Moroccan locust breeding areas across neighboring countries, and the importance of internationally coordinated control efforts.

Worldwide, there is an increasing trend towards adopting biological control methods for managing agricultural pests in order to protect environmental and human health [24,31]. Contemporary biopesticides, developed from bacterial and fungal sources, are designed to selectively target specific pests, locusts included [25], and not to affect non-target organisms such as honeybees or pests’ natural enemies [32]. A prime example of this approach is Somalia’s successful deployment, with the FAO, of the entomopathogenic fungus *Metarhizium acridum* to combat the desert locust during the 2019–2021 upsurge in the Horn of Africa [32]. The fungus’s large-scale effectiveness in controlling both hopper bands and adult swarms demonstrated the feasibility of using environmentally friendly alternatives that are target-specific, unlike broad-spectrum chemical insecticides [32]. Echoing this global shift towards biological control, Tajikistan has also been exploring similar strategies. The Institute of Zoology and Parasitology of the National Academy of Sciences of Tajikistan conducted field trials in the foothills of the Sarsarak ridge in the Dangara district using a biological agent derived from the bacterium *Streptomyces avermitilis,* which produces avermectins, yielding encouraging results [33]. Although *M. acridum* has been officially registered in Kazakhstan and Uzbekistan, its regulatory approval in Tajikistan is pending. In 2024, a national demonstration trial of this biopesticide is organized for its promotion within the framework of FAO’s CCA Locust Programme.

Such initiatives are particularly relevant as locusts (by their nature) and their control present a transboundary challenge. The migratory patterns of locusts do not respect political boundaries, necessitating a collaborative approach to their management. The successful application of biological control in one country can serve as a model for neighboring countries, potentially leading to a coordinated effort across borders to address the locust threat more sustainably. It is worth reiterating that historically, Tajikistan has observed *D. maroccanus* migrations from neighboring countries, underscoring the importance of innovative and collaborative approaches to pest management:

- The southern districts such as Hamadoni, Farkhor, Pyanj, Jayhun, and certain areas of Shahritus have documented locust migrations from Afghanistan.
- The southern districts such as N. Khusrav, Shahritus, and Rudaki have experienced locust migrations from Uzbekistan. The northern districts adjacent to Uzbekistan, including Mastchokh, Zafarobod, and Istaravshan, have also recorded locust immigrations from this country.
- In other northern districts, particularly within B. Gafurov, Konibodom, and Isfara, locust swarm flights have been observed from Kyrgyzstan.

In 2022, a joint initiative involving Tajikistan’s SE-LCE, Uzbekistan’s Ministry of Agriculture, and the FAO identified locust habitats along the Tajikistan–Uzbekistan border. These border regions emerged as primary *D. maroccanus* breeding grounds, with reciprocal transborder locust migrations of hopper bands and adult swarms. Such two-way movements can be influenced by factors like wind patterns and food availability.

As shown in our study, in 2023 the situation escalated, with the SE-LCE reporting an increase in *D. maroccanus* infestations, and consequent crop damage in southern Tajikistan. In response, in April 2023, a joint locust survey was conducted across the Tajikistan–Uzbekistan border. These surveys have been facilitated by the FAO’s CCA Locust Programme. This initiative has consistently enabled regular cross-border surveys, particularly targeting the Sughd region of Tajikistan and the Jizzak region of Uzbekistan in the north, as well as the Khatlon region of Tajikistan and the Surkhandarya region of Uzbekistan in the south. Additionally, in June 2023, separate surveys were carried out in the border areas of Tajikistan and in Afghanistan. Unfortunately, the unstable political situations on the borders with Kyrgyzstan and Afghanistan have hindered cross-border surveys, contributing to cross-border locust migrations.

## 4. Conclusions

The research conducted between 2012 and 2023 reveals a clear trend of increasing locust infestations across Tajikistan, with southern Tajikistan emerging as the most severely impacted region. The species primarily lays eggs in the dry steppes and semi-desert xerophytic areas, favoring the eastern slopes of hills in southern Tajikistan (Khatlon). *D. maroccanus* thrives in semi-desert foothills characterized by a mix of bare compact soil and ephemeral vegetation. This population is supported by the availability of short sedges and grasses (mainly *C. pachystylis* and *P. bulbosa*), which serve as a substrate for egg laying and food sources in these habitats. Our findings reveal that the developmental timeline of this species is subject to annual variability, yet the hatching of first-instar nymphs in the southern regions precedes that in the northern areas by several days or weeks. At the time of writing this manuscript, our latest observation indicated an early hatching in Pyanj, recorded on 23 February 2024. We acknowledge that the absence of meteorological data represents a limitation of this research. Future research should focus on obtaining these data to thoroughly assess the effects of climate change.

Our study shows that *D. maroccanus* has been a serious pest in Tajikistan’s diverse landscapes, affecting pastures, vegetable gardens, and crops in valley, foothill, and hilly areas. Outbreaks of economic importance happened every year within the 12-year period, in all regions, with Khatlon being the most severely affected. The increased scale of control campaigns is notable, from treating 46,500 hectares in 2012 to over 113,681 hectares in 2023. The yearly *D. maroccanus* infestations in Tajikistan underscore the necessity for a unified and proactive approach in pest monitoring and forecasting and early warning/anticipatory action, with a strong emphasis on regional collaboration. Such an approach must include international cooperation for cross-border surveys and the exchange of information and best practices among neighboring countries. However, political instability remains a considerable obstacle to achieving comprehensive success across all borders, highlighting the intricate relationship between environmental management and geopolitical dynamics and the relevance of further support for initiatives such as the FAO CCA Locust Programme.

Another critical factor is the type of insecticides and formulations currently used to control the Moroccan locust. As noted earlier, these are broad-spectrum, water-based chemical insecticides of pyrethroid and organophosphate groups, which are notably harmful for non-target arthropods like honeybees and other beneficial organisms [31,34]. The extensive use of such chemical insecticides, while providing a short-term relief, may have detrimental long-term effects on non-target species, biodiversity in general, and soil and water quality. Considering the extensive use of these chemicals in Tajikistan, with a cumulative area of 943,636 hectares treated against the Moroccan locust between 2012 and 2023, averaging an annual treatment area of 78,636 hectares, the urgency for a paradigm shift towards more sustainable pest management practices is evident. In this context, the adoption of environmentally benign biopesticides that are selective to acridids, such as *M. acridum,* emerges as a compelling alternative. Furthermore, if chemical insecticides are to be applied, Tajikistan urgently needs to switch to ULV application methods. *D. maroccanus* presents a multifaceted transboundary challenge, necessitating coordinated efforts across borders, sectors, and scientific disciplines. Engaging stakeholders across all levels, from local communities, shepherds, and farmers to relevant national and international organizations is needed to curb the agricultural damage caused by *D. maroccanus* as well as ongoing reliance on chemical insecticides. Continued research is critical for advancing sustainable early warning–early action strategies that can respond to current challenges and adapt to future conditions.

## Figures and Tables

**Table 1:** Distinctive features of solitary and gregarious phases in adult Moroccan locusts. Adapted and translated from the recent Moroccan locust monograph [6]. Adapted under Attribution-NonCommercial-ShareAlike 3.0 IGO” (CC BY-NC-SA 3.0 IGO). This translation was not produced by the Food and Agriculture Organization of the United Nations (FAO). FAO is not responsible for the content or accuracy of this translation. The original edition is Russian.

Feature	Solitary Phase	Gregarious Phase
Body Length (mm)	Males: 16.5–22.5 Females: 20.5–28.5	Males: 22.0–28.5 Females: 25.0–38.0
E/F Index *	1.30–1.55	1.50–1.90
Body Coloration	Without orange hue	Orange hue present
Pattern on Hind Femur	With three dark bands	Without dark bands
Tegmina	With numerous distinct dark spots	Completely transparent or with blurred dark spots

* The E/F index is calculated by length of elytra (E) divided by length of femur (F).

## Data Availability

The original contributions presented in the study are included in the article, further inquiries can be directed to the corresponding authors.

## Appendix A

**Table A1:** The timeline of Moroccan locust hatching in southern Tajikistan from 2014 to 2024.

Year	2014	2015	2016	2017	2018	2019	2020	2021	2022	2023	2024
Date of first hatching	20.03	24.03	4.03	6.04	7.03	13.03	3.03	20.02	5.03	6.03	23.02
Districts	Khurasan Kushaniyan	Vakhsh	Dusti Khurasan	Khurasan	Dangara	Dangara	Pyanj Farkhor	Dangara Pyanj	Farkhor	Farkhor	Pyanj

**Table A2:** The timeline of Moroccan locust hatching in central Tajikistan from 2021 to 2023.

Year	2021	2022	2023
Date of first hatching	18.03	14.03	15.03
District	Tursunzoda	Tursunzoda	Tursunzoda

**Table A3:** The timeline of Moroccan locust hatching in northern Tajikistan from 2021 to 2023.

Year	2021	2022	2023
Date of first hatching	12.04	20.04	24.03
District	B. Gafurov	B. Gafurov	Konibodom

**Table A4:** Areas infested by the Moroccan locust in Khatlon region (southern Tajikistan) from 2012 to 2023, with units measured in hectares. Infestation areas higher than 2000 ha are underlined.

District	2012	2013	2014	2015	2016	2017	2018	2019	2020	2021	2022	2023	Average Annual Area	Total for 12 Years
Yavan	600	950	650	320	1100	1355	1000	952	812	596	687	655	806	9677
Khurasan	4200	6000	6000	6700	4700	5496	3500	3500	3520	3600	4160	3455	4569	54,831
Dangara	9000	8300	4430	3900	8370	12,700	11,200	11,195	12,134	16,350	17,865	20,700	11,345	136,144
Levacant	1300	1300	1500	2500	2000	2201	2050	2006	1990	1480	1415	1340	1757	21,082
Kushaniyan	3100	3000	5000	3000	3400	3095	3000	2955	2940	3300	5770	5975	3711	44,535
Vakhsh	3550	5300	3400	2000	4500	2564	3150	3360	5955	7700	7185	7610	4690	56,274
J. Balkhi	3850	4200	1860	1900	3850	3080	2109	2855	3660	6100	5060	5090	3634	43,614
A. Jomi	1200	200	600	300	900	1300	1000	935	830	605	699	760	777	9329
Kabadiyan	1100	2200	2500	4100	1950	6555	3950	3945	3955	3890	4536	2700	3448	41,381
Shahritus	950	1200	3000	4300	2100	4450	2900	2889	2875	3300	3720	3300	2915	34,984
N. Khusrav	1050	650	900	2500	1050	3880	2000	1968	2000	2720	2511	2010	1937	23,239
Dusti	3000	5900	6650	5900	4600	6210	4700	4690	4670	3640	4191	1905	4671	56,056
Jayhun	4200	7500	5200	7800	4500	5635	4200	4401	4200	4570	4880	5065	5179	62,151
Pyanj	4200	5400	2200	2000	4000	6440	4400	12,000	7250	9000	9390	11,145	6452	77,425
Farkhor	3560	5500	2410	2800	4400	8300	4900	5210	4831	5126	5624	7437	5008	60,098
Hamadoni	800	700	850	2000	1000	2570	1600	1560	1305	1105	1262	2260	1418	17,012
Vose	450	900	-	-	-	-	-	-	-	-	-	-	-	1350
Temurmalik	-	-	-	-	-	-	450	460	435	465	527	544	240	2881
Muminobod	-	-	-	-	67	-	-	-	-	-	-	-	-	67
Average area per district	2712	3290	2774	3060	3087	4213	3300	3811	3680	4277	4605	4738		
Total infested area	46,110	59,200	47,150	52,020	52,487	75,831	56,109	64,881	63,362	73,547	79,482	81,951		752,130

**Table A5:** Areas infested by the Moroccan locust area in the Districts of Republican Subordination (central Tajikistan) from 2012 to 2023, with units measured in hectares. Infestation areas higher than 2000 ha are underlined.

District	2012	2013	2014	2015	2016	2017	2018	2019	2020	2021	2022	2023	Average Annual Area	Total for 12 Years
Tursunzoda	2400	3100	2500	4500	2300	3200	3000	3039	3450	4525	4874	4700	3466	41,588
Shakhrinav	2000	1500	1450	2200	2200	2000	1810	1845	1960	2752	3070	3000	2149	25,787
Gissar	2200	1900	720	1800	850	1700	1655	1688	1707	2170	2170	2250	1735	20,810
Rudaki	5800	6700	3732	5000	3200	3800	3330	3393	3350	4298	4601	4375	4298	51,579
Average area per district	3100	3300	2101	3375	2138	2675	2449	2491	2617	3436	3679	3581		
Total infested area	12,400	13,200	8402	13,500	8550	10,700	9795	9965	10,467	13,745	14,715	14,325		139,764

**Table A6:** Areas infested by the Moroccan locust in Sughd region (northern Tajikistan) from 2012 to 2023, with units measured in hectares. Infestation areas higher than 2000 ha are underlined.

District	2012	2013	2014	2015	2016	2017	2018	2019	2020	2021	2022	2023	Average Annual Area	Total for 12 Years
Asht	1063	2115	1660	2310	2260	2840	4000	4244	4105	4530	3778	3210	3010	36,115
Aini	370	-	-	-	-	-	-	-	-	-	-	-	-	370
Gonchi	2623	1990	2010	-	-	-	-	-	-	-	-	-	-	6623
Konibodom	644	700	1250	3220	1830	3220	3000	2997	3470	3745	3770	3650	2625	31,496
Spitamen	805	1305	620	1510	290	1228	1800	1765	2222	2015	2110	1860	1461	17,530
J. Rasulov	895	3644	1830	2150	915	874	1650	1561	1490	1705	1789	1535	1670	20,038
Mastchoh	2060	6200	5700	6750	5312	5300	4633	4898	4975	5805	4722	4740	5091	61,095
Zafarobod	4270	9050	7500	6810	7539	8010	6700	6964	6995	7740	6193	5875	6971	83,646
Isfara	478	645	720	1180	160	1040	4200	3621	3445	3585	3420	3290	2149	25,784
B. Gafurov	2919	7600	6950	11,200	9726	17,740	6800	7055	7185	8485	5691	5430	8065	96,781
Istaravshan	855	1920	2470	1985	420	2562	1500	2474	2240	2485	2200	1870	1915	22,981
Panjakent	1350	750	1020	3080	545	3600	3400	3664	3475	4005	-	-	2074	24,889
Devashtich	-	-	-	-	-	-	-	-	-	-	3649	3670	-	7319
Average area per district	1410	2993	2644	3349	2416	3868	3148	3268	3300	3675	3393	3194		
Total infested area	18,332	35,919	31,730	40,195	28,997	46,414	37,683	39,243	39,602	44,100	37,322	35,130		434,667

**Table A7:** Egg-pod density, nymph and adult density, and abundance of the Moroccan locust in southern Tajikistan. Nymph densities exceeding 5 per 1 m^2^ are considered above the economic threshold.

Survey Area	Year	Coordinates	Altitude (m.a.s.l.)	Egg-Pod Density (per m^2^)	Nymph Count (Specimens)		Adult Count (Specimens)	
					Density per 1 m^2^	Average (Specimens/h)	Density per 1 m^2^	Average: (Specimens/h)
Khurasan	2014	N 38.15490°, E 068.31160°	951				7–185	10,642
	2015	N 37.52229°, E 068.35690°	558	28–46	65–183	11,969	2–25	3246
	2016	N 37.86850°, E 068.59287°	569		7–32	7656		
	2017	N 37.82762°, E 068.58111°	524	230–2400	6–32	7522		
	2018	N 38.15490°, E 068.64160°	832		15–413	12,532		
Dangara	2014	N 37.35272°, E 069.11549°	805				1–5	236
	2016	N 37.82845°, E 069.25731°	802		35–332	14,346		
	2017	N 38.04100°, E 069.25448°	841	56–1000	40–380	12,354		
	2018	N 38.08850°, E 069.26230°	864		32–398	10,986		
	2019	N 38.09353°, E 069.26596°	856		33–1231	12,300	40–1000	14,562
		N 38.11029°, E 069.16424°	1080		13–444	9694		
	2022	N 37.79978°, E 069.23734°	551				15–51	8214
	2023	N 37.98018°, E 069.28860°	617				3–193	6934
Vakhsh	2014	N 37.44100°, E 068.58702°	520	21–37	30–117	7400	1–3	76
	2015	N 37.44110°, E 068.58712°	516	31–40	80–350	17,192		
	2016	N 37.68048°, E 069.05228°	511		12–35	4988		
	2017	N 37.55023°, E 069.19067°	500	35–773	13–35	5486		
	2022	N 37.68050°, E 069.05231°	509	232–308			4–13	3026
Kushaniyan	2015	N 37.48351°, E 068.56143°	559	18–25	93–423	13,435		
J. Balkhi	2016	N 37.25052°, E 069.05031°			10–280	9278		
Kabadiyan	2018	N 37.51311°, E 068.26707°	604	3–8	7–21	2342		
N. Khusrav	2019	N 37.07366°, E 067.91409°	410		1–4	268		
Dusti	2015	N 37.38745°, E 068.26805°	503				1–5	102
	2016	N 37.41087°, E 068.24306°	530		12–126	11,138		
		N 37.41087°, E 068.24300°	523		2–6	432		
	2017	N 37.38745°, E 068.26802°	500	732–1196	10–128	8964		
	2018	N 37.47818°, E 068.41970°	451		13–230	8996		
Jayhun	2014	N 37.22936°, E 068.49013°	530				2–7	100
	2015	N 37.22655°, E 068.51961°	463				5–47	6420
	2016	N 37.39165°, E 068.87559°	454		17–135	11,042		
	2017	N 37.39384°, E 068.87380°	444	140–3416	20–112	6548		
	2022	N 37.39165°, E 068.87559°	450		3–13	1154		
Pyanj	2015	N 37.28065°, E 069.10106°	660				7–15	206
	2016	N 37.38889°, E 068.97417°	430		6–22	490		
	2017	N 37.38885°, E 068.97419°	420	128–140	3–13	564		
	2018	N 37.38889°, E 068.97420°	438		10–130	7984		
	2022	N 37.34275°, E 069.07844°	510		6–43	7645	2–12	3452
Farkhor	2016	N 37.39586°, E 069.31722°	481		35–220	8764		
	2017	N 37.39586°, E 069.31722°	481	120–1297	34–230	9006		
	2022	N 37.34985°, E 069.32135°	566		43–332	14,627	8–43	7002
	2023	N 37.71639°, E 069.38668°	707		7–130	11,734		
		N 37.65116°, E 069.38533°	447				1–43	4023
Hamadoni	2015	N 37.36763°, E 069.35993°	489				2–15	565
	2023	N 37.69886°, E 069.43842°	457				5–232	7323

**Table A8:** Area treated, country aggregate.

Year	Area Treated (ha)
2004	13,000
2005	12,000
2006	8700
2007	60,700
2008	84,000
2009	83,800
2010	73,400
2011	119,800
2012	46,500
2013	84,700
2014	55,400
2015	70,500
2016	35,200
2017	68,000
2018	90,584
2019	80,292
2020	91,856
2021	105,820
2022	101,103
2023	113,681
